# The SELENOT mimetic PSELT promotes nerve regeneration by increasing axonal myelination in a facial nerve injury model in female rats

**DOI:** 10.1002/jnr.25098

**Published:** 2022-06-22

**Authors:** Hugo Pothion, Isabelle Lihrmann, Celia Duclos, Gaëtan Riou, Dorthe Cartier, Loubna Boukhzar, Benjamin Lefranc, Jérôme Leprince, Nicolas Guérout, Jean‐Paul Marie, Youssef Anouar

**Affiliations:** ^1^ Normandie Univ, UNIROUEN, INSERM U1239, Neuroendocrine, Endocrine and Germinal Differentiation and Communication Laboratory Institute for Research and Innovation in Biomedicine (IRIB) Rouen France; ^2^ Normandie Univ, UNIROUEN, UR 3830, Groupe de Recherche sur l'Handicap Ventilatoire et Neurologique Institute for Research and Innovation in Biomedicine (IRIB) Rouen France; ^3^ Fédération Hospitalo‐Universitaire (FHU) Surface Rouen France; ^4^ Normandie Univ, UNIROUEN, INSERM U1234 Institute for Research and Innovation in Biomedicine (IRIB) Rouen France; ^5^ Normandie Univ, UNIROUEN, UMS‐UAR HERACLES, PRIMACEN, Cell Imaging Platform of Normandy Institute for Research and Innovation in Biomedicine (IRIB) Rouen France; ^6^ Otorhinolaryngology and Head Neck Surgery Department Rouen University Hospital Rouen France

**Keywords:** axotomy, facial nerve, motor unit, myelin, peptide, PSELT, RRID:AB_2144666, RRID:AB_2340376, RRID:AB_2340607, RRID:AB_2340812, RRID:AB_2811056, RRID:AB_95186, selenoprotein, SELENOT, therapy

## Abstract

Peripheral nerve injury (PNI) is frequent and many patients suffer lifelong disabilities in severe cases. Although the peripheral nervous system is able to regenerate, its potential is limited. In this study, we tested in a nerve regeneration model in rat the potential beneficial effect of a short mimetic peptide, named PSELT, which derives from SELENOT, an essential thioredoxin‐like selenoprotein endowed with neuroprotective and antioxidant activities. For this purpose, the right facial nerve of female Long–Evans rats was axotomized then bridged with a free femoral vein interposition graft. PSELT (1 μM) was injected into the vein immediately and 48 h after the injury, and the effects observed were compared to those found after an end‐to‐end suture used as a gold standard treatment. Whisking behavior, electrophysiological potential, and histological analyses were performed 3 months after injury to determine the effects of these treatments. These analyses revealed that PSELT‐treated animals exhibit a better motor recovery in terms of protraction amplitude and velocity of vibrissae compared to control and end‐sutured nerve animal groups. Moreover, administration of PSELT following injury enhanced muscle innervation, axonal elongation, and myelination of newly formed nerve fibers. Altogether, these results indicate that a PSELT‐based treatment is sufficient to enhance facial nerve myelination and regeneration and could represent a new therapeutic tool to treat PNI.

## Graphical abstract

We demonstrate that administration of a novel selenopeptide named PSELT to a lesioned facial nerve improved its regeneration and ameliorated motor performances in rat, strongly suggesting that PSELT is a valuable therapeutic candidate to treat peripheral nerve injuries.

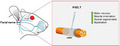

**Abbreviations**
EMG: electromyogram
ER: endoplasmic reticulum
ES: epineural suture
MUAP: motor unit action potential
NF200: neurofilament 200 KDa
P0: myelin protein zero
PNI: peripheral nerve injury
PNS: peripheral nervous system
PSELT: SELENOT mimetic peptide
ROS: reactive oxygen species
SC: Schwann cells

**Significance**

Peripheral nerve injury is a frequent condition whose severity depends on the nerve involved. Efficient treatments that ensure full functional recovery are yet to be identified. Here, we demonstrate that administration of a novel selenopeptide named PSELT to a lesioned facial nerve improved its regeneration and ameliorated motor performances in rat. Together, our data strongly suggest that PSELT is a valuable therapeutic candidate to treat peripheral nerve injuries.

## 1 INTRODUCTION

It is well known that contrary to central nervous system axons, those of the peripheral nervous system (PNS) have an intrinsic regeneration ability. However, complete recovery is not observed in case of severe peripheral nerve injury (PNI), which represents a major source of disability. Nowadays, microsurgery suture is still the gold standard and no other treatment is available to repair this lesion (Faroni et al., 2015). In order to strengthen the intrinsic regenerative capacities of peripheral nerves, new potential therapeutic strategies have emerged based on neurotrophic factors, hormones, or pharmacological compounds (Catrina et al., 2013; Hart et al., 2004; Lu et al., 2018). Although these approaches improve somewhat the recovery, outcomes are comparable to those seen after microsurgery repair. Thus, investigations are still necessary to uncover new treatments that could ensure a better nerve recovery.PNI induces ischemia when the blood–nerve barrier is altered resulting in local hypoxia (Weerasuriya & Mizisin, 2011). It has been shown that hypoxia and reoxygenation and the resulting reactive oxygen species (ROS) could affect the survival and neurotrophic effects of primary cultured Schwann cells (SCs) (Zhu et al., 2010). These cells play a crucial role in PNI since they phagocyte cellular and myelin debris through Wallerian degeneration and promote the elongation of re‐growing axons through secretion of neurotrophic factors (Zhu et al., 2010). Although preclinical studies using pharmacological agents targeting ROS have shown promising outcomes to repair peripheral nerves (Caillaud et al., 2018; Kamboj et al., 2010; Saini et al., 2007; Sharma & Sayyed, 2006), to our knowledge most attempts to translate to the clinic therapeutics based on antioxidants such as vitamins C and E, and glutathione or some elements such as zinc have been unsuccessful (Dao et al., 2015; Ghezzi et al., 2017). Recently, it was shown that treatment with selenium has a beneficial effect in an acute nerve injury model in rat (Zendedel et al., 2017). Selenium is known to be incorporated in a particular class of proteins named selenoproteins defined by the presence of at least one selenocysteine (Sec), a selenium‐containing amino acid, into their polypeptide chain. Endowed with a higher nucleophilicity than cysteine, Sec confers to selenoproteins a better redox resistance to irreversible inactivation by overoxidation, making of these proteins one of the main antioxidant family (Dardashti et al., 2016). Among the most conserved selenoproteins, we have previously shown that SELENOT exerts antioxidant and neuroprotective effects (Anouar et al., 2018; Boukhzar et al., 2016). We have therefore developed a SELENOT‐derived peptide (named PSELT) which contains the active redox center CVSU, in order to use it as a short mimetic of the protein for potential therapeutic applications. This peptide proved to be effective in an ischemia/reperfusion injury model in adult rat heart ensuring a better cardiac contracture, a reduction of infarct size, and a decrease in the levels of ROS and caspase 3 activity (Rocca et al., 2018). Moreover, PSELT protected dopaminergic neurons and fibers of the nigrostriatal pathway, resulting in improved motor activity in mice exposed to the neurotoxin MPTP (1‐methyl‐4‐phenyl‐1,2,3,6‐tetrahydropyridine). Higher cell survival, and lower ROS levels and caspase 3 activity were observed in PSELT‐treated dopaminergic neurons compared to controls (Alsharif et al., 2021).

Despite the vast literature reported on the beneficial effects of antioxidant enzymes including selenoproteins in various pathologies, studies on their advantageous impact in PNI are scarce (Amato et al., 2019; Mantovani et al., 2003; Valentine et al., 2008). Therefore, we tested in the present study the hypothesis that local application in the lesion site of PSELT may increase SC integrity and nerve regeneration in a facial nerve axotomy model. Our data showed that 3 months after injury, PSELT‐treated rats exhibit better motor performances and enhanced nerve myelination and regeneration observed at the electrophysiological and histological levels.

## 2 MATERIALS AND METHODS

### 2.1 PSELT peptide

The SELENOT‐derived peptide 43–52 (PSELT) was designed as previously reported (Rocca et al., 2018). The peptide (FQICVSUGYR, U = Sec) was synthesized on a Liberty Microwave assisted automated peptide synthesizer (CEM, Saclay, France) and purified by HPLC on a 21.2 × 250 mm Jupiter C18 (5 μm, 300 Å) column (Phenomenex, Le Pecq, France) at a purity grade higher than 98.6% as previously described (Touchard et al., 2020). The authenticity of the synthetic peptide was controlled by MALDI‐TOF‐MS on an UltrafleXtreme (Bruker Daltonik, Bremen, Germany) in the reflector mode with α‐cyano‐4‐hydroxycinnamic acid as a matrix.

### 2.2 Surgical procedure

The animal protocol was designed to minimize pain or discomfort to the animals. All experimental procedures were in accordance with the European Community guiding principles on the care and use of animals (86/609/CEE; Official Journal of the European Communities no. L358; December 18, 1986), French Decree no. 97/748 of October 19, 1987 (Journal Officiel de la République Française; October 20, 1987), and the recommendations of the Cenomexa ethics committee (#9166).A total of 27 female Long–Evans rats aged 8 weeks were used in this study. During all the surgical procedures, animals were sedated with isoflurane (2.5%). The right facial nerve was exposed via a 4‐cm skin incision and transected using micro‐scissors. After the nerve axotomy, different procedures were carried out depending on the animal group:

- Epineural suture group (ES group) (*n* = 10): the two segments were closed directly by an epineural suture with 10–0 microsurgical sutures.
- Control group (*n* = 10): the two segments were bridged with an extract of the right femoral vein (6 mm) removed from the same animal leaving a 3 mm gap between the two nerve segments. Vein–nerve suture was performed with 10–0 microsurgical sutures. Ten microliters of Matrigel® were gently injected in the 3 mm gap of the femoral vein with a tip diameter of 72 μm mated to a 25‐μl Hamilton syringe (Hamilton Company).
- PSELT group (*n* = 7): the two segments were bridged and the rats received a first injection of 10 μl of Matrigel® supplemented with PSELT at the concentration of 1 μM. Forty‐eight hours later, the injury site was exposed again and a second administration of PSELT at 1 μM diluted in physiologic serum was performed.

The surgery wound was sutured with 4–0 surgical sutures after Matrigel® injection in vein.

Before and after the experiments, all rats were kept on standard laboratory chow and tap water ad libitum in cages with room temperature maintained at 21 ± 0.5°C, with a hygrometry maintained at 60 ± 10% and a lighting condition of 12 h light/dark cycle. Rats were housed in groups of five animals, and had access to a cardboard tube and a plastic house. Cages were cleaned twice a week. Video recording and motor unit action potential measurements have been performed 90 days after surgery (*n* = 6–8 per group), whereas histological analyses have been conducted 90 days after surgery on four to seven animals per group, in order to be able to conduct additional experiments if needed.

### 2.3 Video recording

**FIGURE 1:**
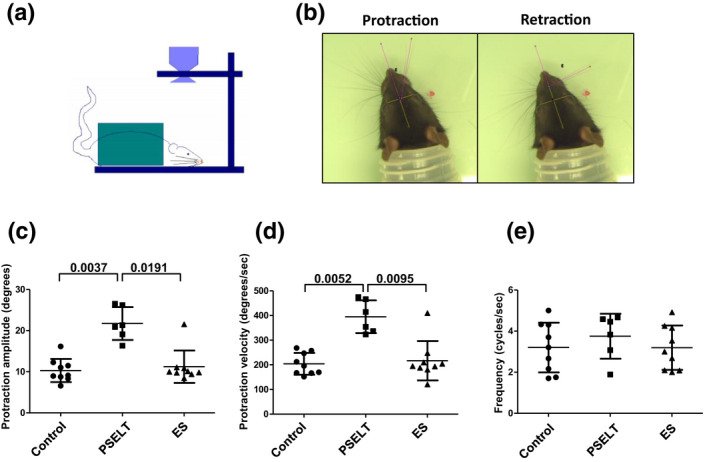
Effect of PSELT‐based therapy on facial nerve motor performance recovery 12 weeks after injury. (a) Schematic representation of the experimental procedure. The camera is perpendicular to the immobilized rat and records the vibrissae movements. (b) Video recording allows precise measurement of protraction angle, protraction velocity, and mean frequency of the whisker movement on the operated side. The protraction amplitude is obtained by the difference between the protraction angle and the retraction angle. Note that no difference in whisking was observed in the intact side. (c–e) Effect of PSELT on whisking performance. Protraction amplitude (c), protraction velocity (d), and mean whisking frequency (e) were compared after injured facial nerve was bridged with vein (control) or with vein infused with PSELT, or was treated with the standard surgery procedure (ES: epineural suture). Video recordings were analyzed with Image J Software. *N* = 9 animals for Control and ES groups, and *N* = 6 for PSELT group. Measurements are expressed as average ± standard deviation. Statistical evaluations were based on Kruskal–Wallis test.

Three months following injury, two large vibrissae of the C row (C3 and C4) on the injured side of the face were used for biometric analysis under light anesthesia. All other vibrissae of the injured side were cut using small fine scissors, then the rats were inserted into a rodent restrained. Using CM_Blr1300‐75 g (Biometrics, Gometz‐le‐Châtel, France), whisking behavior was video recorded for 6–10 min during active exploration. Video images of whisking behavior were sampled at 80 Hz and the video camera shutter was open for 4 ms. Recorded video sequence fragments were selected for analysis according to the stable position of the animal's head and large‐amplitude vibrissae sweeps. The tip of the rat's nose and the inner angles of both eyes were defined as reference points. Vibrissae were represented by a straight line passing between C3 and C4. Frame‐by‐frame analysis using ImageJ software evaluated protraction amplitude, amplitude velocity, and the whisking frequency. Protraction has been assessed by the rostral angle between the midsagittal plane and the whisker shaft (Figure 1b).

### 2.4 Motor unit action potential measurements

Electromyogram evaluation of the facial nerve was performed 3 months after surgical procedure. Animals were anesthetized with intraperitoneal injection of ketamine (60 mg/kg) and xylazine (7.5 mg/kg.) then the rat's right facial nerve was exposed. A bipolar stimulating electrode and two unipolar platinum recording electrodes were used to induce and record electrical activity, respectively. The stimulating electrode was placed under the proximal facial nerve (at the exit point of the stylomastoid foramen before the site of injury) and the recording electrodes were placed within the mystacial pad (row C). The evoked action potential in responding to the stimuli (500 ms, 1 V, 250 Ω) in the ipsilateral facial nerve was recorded using PowerLab 26T and analyzed with LabChart 7 Pro software (Sydney, Australia).

### 2.5 Histological evaluation

Immediately following electromyogram evaluation, rats were perfused and tissues were fixed through 4% paraformaldehyde perfusion. The right facial nerve and the right mystacial pad were dissected then postfixed overnight in the same fixative at 4°C and immersed in 30% sucrose‐PBS 24 h at 4°C. Samples were embedded in optimal cutting temperature compound (ThermoFischer Scientific) and cut with a cryostat microtome (Leica). Sections were incubated with primary antibodies (RRID:AB_2811056: S100, Dako, Z0331, RRID:AB_2144666: P0, Novusbio, NB100‐1607, RRID:AB_95186: neurofilament 200 KDa, Millipore, MAB5262) diluted in blocking solution (PBS containing 10% normal donkey serum and 0.3% Triton X‐100) overnight at 4°C. Sections were washed 10 min in PBS three times, then stained with Alexa Fluor 488, 594 or 647‐conjugated secondary antibodies (1:500; RRID:AB_2340607: Jackson ImmunoResearch, 711‐175‐152, RRID:AB_2340812: Jackson ImmunoResearch, 715‐165‐140 and RRID:AB_2340376: Jackson ImmunoResearch 703‐546‐155) diluted in blocking solution for 60 min at room temperature. Sections were washed 10 min in PBS three times before coverslips were applied with Mowiol® 4‐88 (Biovalley, Nanterre, France). Images were acquired on a photonic fluorescence microscope (Zeiss).

For morphometric analysis, axonal counting and axonal myelination assessment, 30 μm transverse nerve sections were done along the lesion site with cryostat microtome (Leica). Nerve diameter measurement corresponds to the average of 8 fiber diameters assessed on the same nerve section. Measurements have been conducted at the distal part of the nerve on three consecutive sections. After staining with antibodies against chicken anti‐P0 (1:200, USBiological Life Sciences, Swampscott, MA, USA), rabbit anti‐S100 (1:200, Dako) and mouse anti‐neurofilament 200 KDa (1:500, Merck Millipore), five areas of 5 mm^2^ were randomly selected and analyzed with ImageJ software.

### 2.6 Study limitations

Although the same number of animals (10) was initially included in each group for this study, the intrusive nature of the surgery for nerve end suture (ES group), femoral vein–nerve end suture, and repeated peptide or vehicle administration (PSELT and controls) led to a disparity in this number between the groups due to animal mortality, with a higher mortality in control and PSELT groups after surgery and motor performance experiment. For electromyography, results from six to eight animals could be exploited given the quality of the electromyograms. It is important to note that for electromyography recordings, nerve stimulation has been performed using a hook nerve stimulator which can further damage the injured nerves. For IHC studies, nerves from four to seven animals were analyzed. The number of animals included in each analysis is reported in Table 1.

**TABLE 1:** Animal groups and number of animals in each group

Number of animals						
Time point	12 weeks					
Experimental conditions	Surgery	Motor performance	Electromyography	IHC facial nerve		
				NF200	NF200/S100	NF200/P0
ES	10	9	8	7	4	4
Control	10	9	6	6	5	5
PSELT	7	6	6	6	6	6

Abbreviation: IHC, immunohistochemistry.

### 2.7 Statistical analysis

All data are presented as means ± standard deviation (SD). Shapiro tests were performed for assessing data distribution. These tests revealed that our data are not normally distributed. Therefore, non‐parametric tests have been performed. Comparison of medians was performed using Kruskal–Wallis tests followed by a Dunn post‐tests. All the statistical tests have been performed using Graphpad Prism 8 (GraphPad Software). In all tests, a value of *p* < .05 was considered statistically significant. Exact *p* values are shown in all the figures.

## 3 RESULTS

### 3.1 Motor performance recovery 12 weeks after axotomy

In order to investigate the potential therapeutic benefit of PSELT in PNI, a Matrigel solution containing PSELT at the concentration of 1 μM (PSELT group) or Matrigel alone (control group) were injected into the space left between the two nerve stumps within the vein that bridged the transected facial nerve of rats. After 48 h, the injured facial nerve was exposed again and a second injection of PSELT at the same concentration or Matrigel® was performed. In the perspective of a clinical application, these two experimental groups were compared to rats in which both nerve stumps were bridged by an epineural suture (ES group), a microsurgery procedure used in clinical practice. Three months after injury, rats were placed in a restraint system in order to evaluate the effect of PSELT on motor recovery (Figure 1a). To evaluate the effect of PSELT on motor recovery of the transected facial nerve, we assessed vibrissae movement using video recording (Figure 1b). Analysis of video recordings showed that early administration of PSELT at the concentration of 1 μM at day 0 and day 2 post‐injury significantly increases functional motor performance recovery illustrated by an elevation of protraction amplitude and protraction velocity in the PSELT group (21.74 ± 1.99° and 394.83 ± 33.23 °/s, respectively, *n* = 6) compared to the control group (10.30 ± 1.41° and 203.94 ± 22.06 °/s, *p* = .0037 and .0052 respectively, *n* = 9) and to the ES group (9.94 ± 0.41° and 192.44 ± 17.74 °/s, *p* = .0191 and .0095, respectively, *n* = 9) (Figure 1c,d). However, no difference was observed for the average frequency (Figure 1e). These data show that early PSELT administration improves motor function restoration after PNI.

### 3.2 Motor unit functionality 12 weeks after axotomy

**FIGURE 2:**
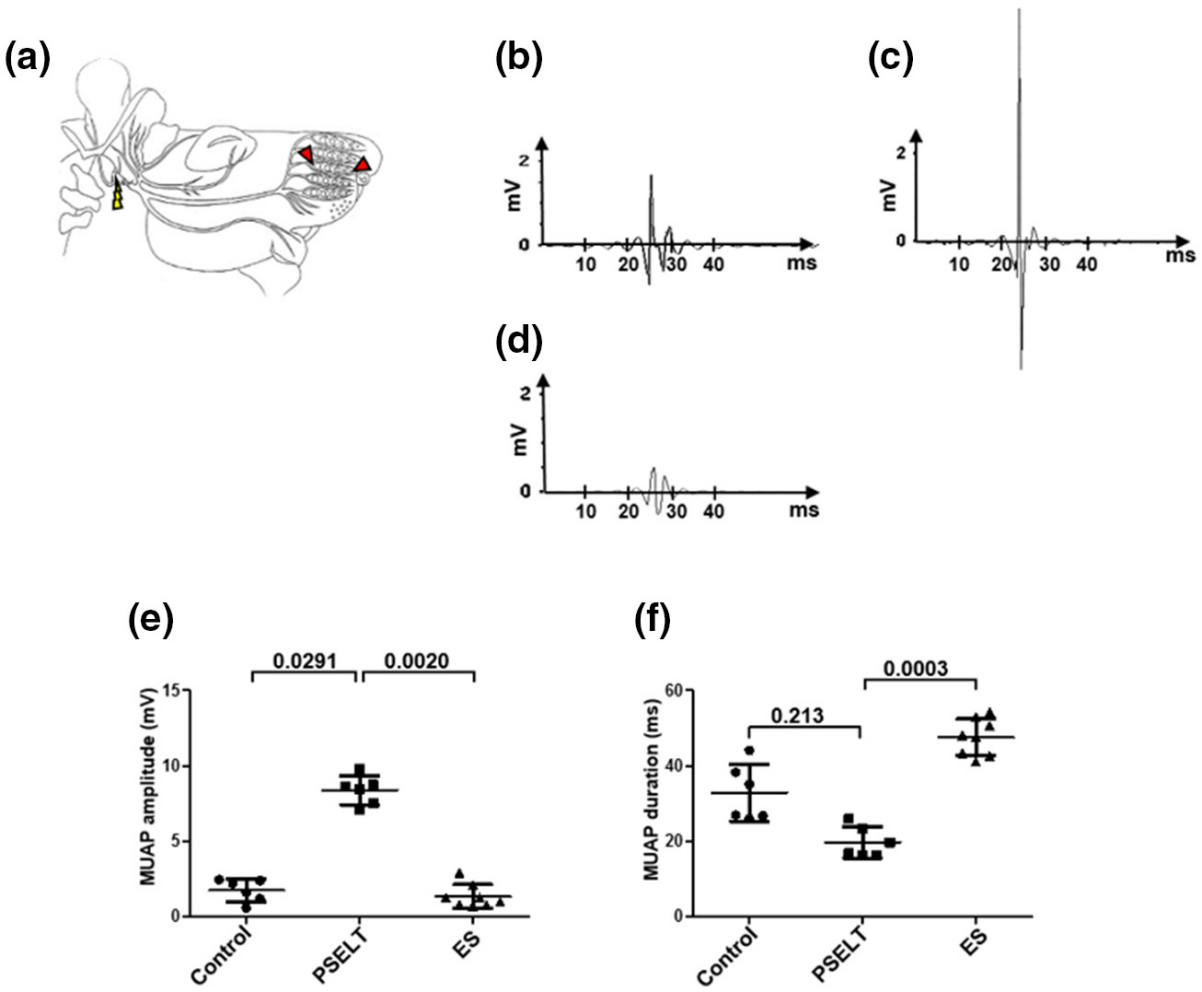
Effect of PSELT‐based therapy on motor end plate innervation 12 weeks after injury. (a) Schematic drawing of the experimental procedure. On the operated side, the facial nerve was exposed, and 1‐mV current was applied by an electrode (yellow thunderbolt) upstream of the injury. Evoked action potentials were recorded with two electrodes (red triangles) in mystacial pad (row C). (b–d) Motor unit action potentials (MUAP) were recorded 3 months after injury in the control group (b), in rats treated with PSELT (c) and in surgically treated rats (d). (e,f) Effect of PSELT on amplitude (e) and duration (f) of MUAP was analyzed using LabChart 7 Pro software. *N* = 6 animals for Control and PSELT groups, and *N* = 8 for ES group. Measurements are expressed as average ± standard deviation. Statistical evaluations were based on Kruskal–Wallis test.

To explain these motor performance improvements in the PSELT group, we hypothesized that nerve regeneration was ameliorated in the PSELT group compared to the other groups. Therefore, nerve regeneration was assessed by electromyographic analysis, notably motor unit action potential (MUAP) evaluation. Usually, axotomy disrupts MUAP wave in terms of amplitude, duration, and shape. To assess these parameters in our facial nerve model, a stimulus of 1 mV during 500 ms was applied at the stylomastoid foramen exit upstream of the injury site and the MUAP was recorded with two electrodes placed on the mystacial pad (row C) (Figure 2a). MUAP recordings highlighted a better outcome in the PSELT group (Figure 2b–d). Indeed, the PSELT group had a higher average MUAP amplitude (8.32 ± 0.48 mV, *n* = 6) than the control group (1.73 ± 0.38 mV; *p* = .0291, *n* = 6) or the ES group (1.34 ± 0.39 mV; *p* = .0020, *n* = 8) (Figure 2e). This better nerve recovery was also observed for the MUAP duration with a shorter duration in the PSELT group (19.65 ± 2.06 ms) compared to the ES group (47.55 ± 2.44 ms; *p* = .0003), and a decrease tendency compared to the control group (32.84 ± 3.75 ms; *p* = .213) (Figure 2f) although no statistical difference was observed with the latter group. Overall, these data indicate that PSELT improves nerve functional regeneration 12 weeks after its administration into the injury site.

### 3.3 Histological outcome of facial nerve regeneration 12 weeks after axotomy

**FIGURE 3:**
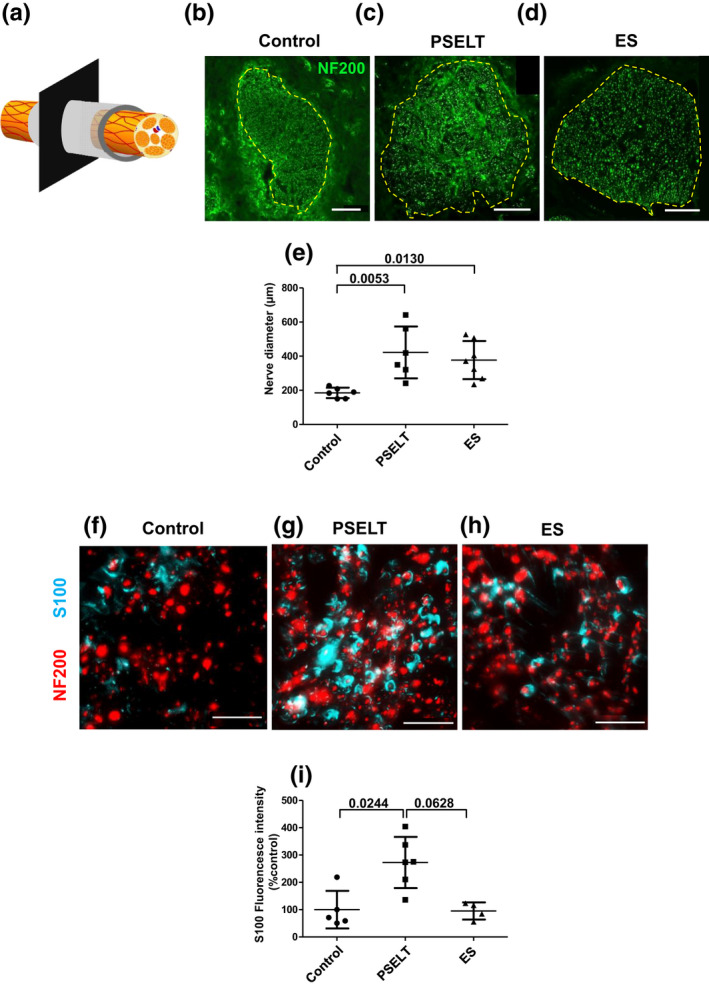
Effect of PSELT‐based therapy on histological repair of the facial nerve 12 weeks after injury. (a) Schematic drawing representing the image acquisition procedure. Transverse sections were performed in the injury site about 0.1 mm beyond the proximal stump toward the distal direction. (b–d) Confocal imaging shows labeling of the neurofilament marker NF200 (green) in transverse sections of the facial nerve from the control, PSELT and ES groups 3 months after injury. The dotted line indicates the perineurium. *Scale bar*: 100 μm. (e) Nerve diameters were assessed from confocal images using Image J software (*n* = 6–7). (f–h) Three months after injury, transverse sections were stained with antibodies against the axonal marker NF200 (red) and the SC marker S100 (blue). *Scale bar*: 25 μm. (i) S100 fluorescence intensity in the PSELT and ES groups were determined and compared to the control group. Fluorescence quantification was performed using Image J software. *N* = 6 animals for Control and PSELT groups, and *N* = 7 for ES group for axonal diameter (e) and *N* = 5 animals for Control, *N* = 6 animals for PSELT groups, and *N* = 4 animals for ES group for S100 fluorescence intensity (i). Measurements are expressed as average ± standard deviation. Statistical evaluations were based on Kruskal–Wallis test.

Twelve weeks after injury, facial nerves were taken and transversal sections were performed at about 100 μm downstream of the proximal stump (Figure 3a). Nerve integrity was examined in these sections after immunostaining with the neurofilament marker NF200 (RRID:AB_95186, neurofilament 200 KDa, Millipore, MAB5262) (Figure 3b–d). While in a normal nerve, regular myelinated fibers are organized in fascicules (Gao et al., 2013), all our experimental groups exhibited numerous irregular and thin nerve fibers homogeneously distributed throughout the entire field, typical of a regeneration process. Moreover, a smaller diameter of the damaged nerve segment was observed in rats that received vehicle injection (Matrigel®) compared to the others, with a mean diameter of 184.95 ± 15.33 μm for controls (*n* = 6) and 422.25 ± 75.99 μm for the PSELT group (*n* = 6) (*p* = .0053) or 377.19 ± 55.84 μm (*p* = .0130) for the ES group (*n* = 7). Of note, the difference between the PSELT and the ES groups did not reach statistical significance (*p* > .9999; Figure 3e).

It is now well established that SC have a crucial role in peripheral nerve repair. In order to investigate the effect of the PSELT peptide on SC occurrence, transverse sections were stained with antibodies against the SC marker S100 (RRID:AB_2811056: S100, Dako, Z0331) (Figure 3f,g). The S100 fluorescence intensity was quantified in five areas of 5 mm^2^ of the nerve section for each animal to take into account nerve heterogeneity. This analysis revealed that rats that received PSELT (*n* = 6) exhibit a higher mean fluorescence intensity of S100 compared to control rats (*n* = 5) (+172%; *p* = .0244) and to rats with ES (*n* = 4) (*p* = .0628). Note that there is no difference between the ES animals and the control group for S100 labeling (Figure 3I).

### 3.4 Influence of PSELT‐based therapy on myelination of regenerated axons 12 weeks after axotomy

**FIGURE 4:**
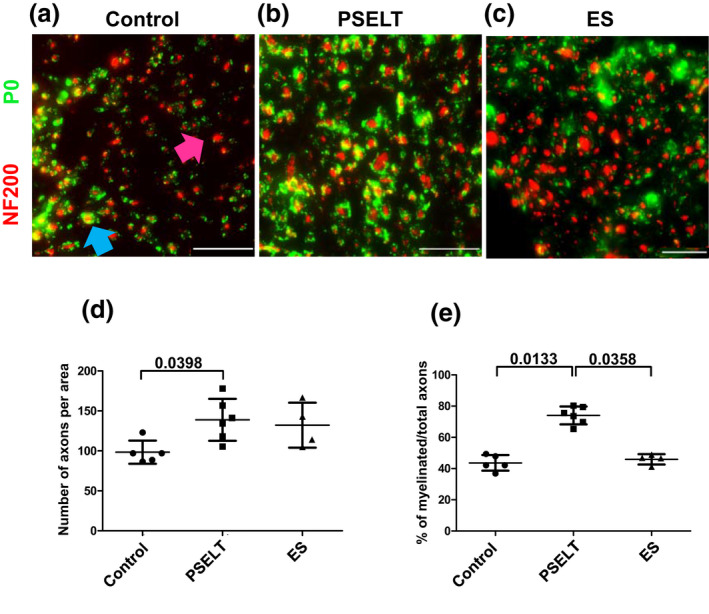
Effect of PSELT‐based therapy on myelination of facial nerve 12 weeks after injury. (a–c) Transverse sections performed in the injury site about 0.1 mm beyond the proximal stump toward the distal direction were stained with antibodies against NF200 (red) and the myelin marker protein zero (P0) (green). Blue and pink arrows indicate a myelinated and an unmyelinated nerve fiber, respectively. *Scale bar*: 25 μm. (d) Axon number counting was performed using Image J Software. Counting was repeated five times for every animal to account for nerve heterogeneity and averages are presented. (e) Myelinated axon number counting was performed using Image J Software. Counting was repeated five times for every animal and averages are presented. *N* = 5 animals for Control group, *N* = 6 animals for PSELT group and *N* = 4 for ES group. Measurements are expressed as average ± standard deviation. Statistical evaluations were based on Kruskal–Wallis.

After cleaning of the lesion site, SC participate in axon elongation through neurotrophic factor production and secretion, and then in myelination of newly formed axons. Thus, we asked whether the higher S100 fluorescence intensity observed in rats that received PSELT, could be related to a better axonal regrowth and myelination. To address this question, transverse sections of the distal stumps of facial nerve were stained with antibodies against the axon‐abundant neurofilament marker NF200 and the protein zero (P0) (RRID:AB_2144666: P0, Novusbio, NB100‐1607), a major structural protein of the peripheral myelin sheath. In line with data in the literature, immunofluorescence analyses showed the occurrence in the lesion site of axons wrapped or not by a myelin sheath (Figure 4a–c). In agreement with the S100 fluorescence intensity results, counting of myelinated and unmyelinated axons (Figure 4a, blue and pink arrows, respectively) was performed in five areas of 5 mm^2^ for each section. The axonal density was higher in the PSELT (*n* = 6) and ES groups (*n* = 4), with an estimated number of axons by area of 139 ± 13 and 132 ± 14, respectively, whereas the control group (*n* = 5) had a mean of 98 ± 7, which is statistically different from that of the PSELT group (*p* = .0398) (Figure 4d). Moreover, we found twice as many myelinated axons in the PSELT group (74.03 ± 2.86) compared to the control (43.66 ± 2.52; *p* = .0133) and ES (45.89 ± 1.65; *p* = .0358) groups (Figure 4e). Altogether, these data indicate that a PSELT‐based therapy could promote sustained regenerative sprouting and improve myelination of newly formed axons.

## 4 DISCUSSION

This study builds on previous work in which PSELT exhibited a protective effect against neurodegeneration in a Parkinson's disease model (Alsharif et al., 2021), and after ischemia/reperfusion injury (Rocca et al., 2018). We investigated here the therapeutic potential of the PSELT peptide in a facial nerve defect, a well‐documented model to evaluate PNI. We demonstrated that early injection of PSELT enhances nerve regeneration in terms of improved motor performances of the whisker pad and higher myelination of the regrown axons in the injured facial nerve.

PSELT‐treated animals showed motor restoration as revealed by a protraction amplitude and a protraction velocity that are almost twofold higher than those assessed for untreated or gold standard‐treated rats. This improvement in motor recovery was supported by electrophysiologic analysis of MUAP which evaluates the neuromuscular system activity and reflects the structure of a motor unit (Bischoff et al., 1994). In axotomy cases, axonal regeneration provokes the appearance of polyphasic MUAP with small amplitude referred to as “nascent potentials” which indicate weak reinnervation (Crone & Krarup, 2013; Robinson, 2000). Unsurprisingly, untreated and gold standard‐treated rats showed a poor restoration of MUAP as reported previously (Guntinas‐Lichius et al., 2002; Hadlock et al., 2010; Tomov et al., 2002). However, PSELT‐treated rats had a higher amplitude and a shorter duration compared to the other experimental groups and had mostly a biphasic or triphasic shape in a proportion similar to that seen in healthy rats. Because the amplitude depends on various factors such as the number of axons responding to the stimulus, the synchronization of their response and the size of the motor unit, an elevation of this parameter indicates a higher number of nerve fibers recruited and their better remyelination, indicating thereby a better nerve regeneration (Archibald et al., 1991). Indeed, ES group presents shorter EMG amplitudes in comparison to PSELT group although the number of axons was comparable to that seen in the PSELT group. However, ES group presents a lower number of S100‐positive cells and a smaller ratio of P0‐positive fibers into the facial nerves in comparison to PSELT group. These results demonstrate that in ES group there is a high number of axons into the facial nerve but that these fibers are not myelinated (lower P0 staining) and are not associated with Schwann cells (lower S100 staining). The small number of myelinated fibers can explain the weak functional recovery observed and the short amplitudes of the electrophysiological measurements. Compared to other therapeutic strategies tested, the improvement observed in this study with PSELT is at least similar to those reported following neurotrophic factor administration or inhibition (Barras et al., 2009; Guntinas‐Lichius et al., 2005). These data show that administration of PSELT at 1 μM given twice to rats early after nerve injury was sufficient to ameliorate regenerated nerve functioning. Nevertheless, additional adjustments are still necessary to determine the optimal dose, duration, and route of administration that could further improve the therapeutic effect of the peptide.

Few hours following injury, it is well known that the proximal stumps of peripheral axons undergo changes at their tips where numerous axonal branches emerge from the growth cone which begins to elongate. In agreement, our analysis of the injury site revealed histological features of regenerative processes in all experimental groups with changes in the architecture of the fascicules which are numerous, irregular, and comprising some thinner axons, compared to controls. The PSELT and ES groups showed the highest axonal density compared to the control group. However, the proportion of myelinated axons, which were less clustered, was increased to a similar level in rats treated with PSELT compared to the other groups, suggesting a more advanced nerve recovery in the former groups. These observations are in line with the video recordings and electromyogram results and suggest that PSELT impacts the sprouting and/or elongation processes. Further investigations are required to analyze the impact of PSELT on axonal sprouting particularly in terms of synkinesis, one of the major issues following facial nerve regeneration (Çelik et al., 2000).

Rocca et al. demonstrated that the PSELT‐induced cardioprotective effect relies on the activation of RISK pathway through PI3K‐Akt and ERK1/2, and requires mitoKATP‐channel opening (Rocca et al., 2018). Interestingly, recent studies indicated that facial axotomy triggers simultaneous activation of ERK/MAPK and PI3K/AKT signaling in facial nerve cells and axons from day 1 following axotomy, and that this activation promotes neuron survival and axon regrowth (H. Huang et al., 2017; H.‐T. Huang et al., 2017). Further studies showed that both pathways have complementary functions: ERK pathway activation promotes nerve fiber plasticity and elongation, while Akt pathway signaling through PI3 kinase activation at the level of SC has a crucial role in axon ensheathment and regulation of myelin sheath thickness in the PNS via increased myelin‐binding protein and P0 levels (Domènech‐Estévez et al., 2016; Hausott & Klimaschewski, 2019). As our results showed a higher myelination after PSELT administration, these data suggest that PSELT could act in facial axotomy/regeneration in a similar manner to that observed in ischemia/reperfusion injury through activation of the same signaling pathways. Following PNI, injured motor neurons undergo significant changes in their metabolism, morphology, and electrophysiology (Graeber, 1994). These changes in neural cells promote ROS formation (Llobet Rosell & Neukomm, 2019; Rodella et al., 2016), which is reduced by various antioxidant molecules whose expression is stimulated in motor neurons as demonstrated for glutathione reductase, thioredoxin 1, and the selenoprotein thioredoxin reductase 1 in a hypoglossal nerve injury model in rats (Hama et al., 2010; Mansur et al., 1998). In fact, PSELT may balance ROS and nitric oxide levels to maintain them at sustainable levels for SC, thus enabling these cells to release neurotrophic factors necessary for the regeneration of the injured nerve, as previously suggested also for selenium or ascorbic acid (Huff et al., 2020; Kizilay et al., 2017). This potential pro‐survival antioxidant effect of PSELT could be carried by the trace element selenium present in PSELT, which was recently shown to exert a protective effect against ferroptosis in neural tissue (Alim et al., 2019; Ingold et al., 2018). In a similar manner to the selenocysteine‐containing peptide investigated by Alim et al., PSELT could prevent axonal damage by inhibiting ferroptosis although this cell death process has not been established yet in peripheral neuropathy. It should be mentioned that PSELT is able to cross the plasma membrane in vitro and in vivo and to target different pathways in neuronal cells (Alsharif et al., 2021). Many studies pointed out the crucial role of the immune system in the process of neurodegeneration/regeneration after PNI (Gaudet et al., 2011; Greathouse et al., 2016; Quan & Gao, 2006). In the present study, PSELT was administered into the injury site, and therefore we can assume that the protective action of the peptide applies to all cells present in the injury site, including activated immune cells. The peptide could then decrease the inflammation associated with injury/transplantation as was previously observed after administration of Tempol (4‐hydroxy‐2,2,6,6‐tetramethylpiperidine‐N‐oxyl), a superoxide dismutase mimetic, in mouse models of CNS inflammatory injury (Bernardy et al., 2017). Further investigations are required to identify the mechanism of action of PSELT in facial nerve regeneration and to elucidate whether PSELT, like its precursor SELENOT, acts at the level of the ER to accompany nerve regeneration (Dikiy et al., 2007; Hamieh et al., 2017; Pothion et al., 2020).

## 5 CONCLUSION

In conclusion, the present study demonstrated the beneficial effect of PSELT in facial nerve injury/regeneration. Its administration in the lesion site following injury results in better functional recovery of the nerve compared to other treatments of PNI. Consistent with its effect in motor restoration, PSELT enhances nerve regeneration through increased axonal regrowth and myelination, thus identifying PSELT as a new therapeutic candidate to treat PNI.

## AUTHOR CONTRIBUTIONS

*Conceptualization*, Y.A., J.‐P.M., I.L., N.G., and H.P.; *Methodology*, Y.A., J.‐P.M., I.L., N.G., H.P., and J.L.; *Investigation*, H.P., I.L., N.G., C.D., D.C., G.R., and L.B.; *Format Analysis*, Y.A., J.‐P.M., I.L., N.G., and H.P.; *Validation*, Y.A., J.‐P.M., I.L., N.G., and H.P.; *Visualization*, Y.A., J.‐P.M., I.L., N.G., and H.P.; *Writing – Review & Editing*, Y.A., J.‐P.M., I.L., N.G., and H.P.; *Funding Acquisition*, Y.A. and J.‐P.M.; *Supervision*, Y.A., J.‐P.M., I.L., and N.G.

## CONFLICT OF INTEREST

The authors declare that they have no competing interests.

## DECLARATION OF TRANSPARENCY

The authors, reviewers and editors affirm that in accordance to the policies set by the *Journal of Neuroscience Research*, this manuscript presents an accurate and transparent account of the study being reported and that all critical details describing the methods and results are present.

## CONSENT FOR PUBLICATION

All authors have read the manuscript and indicated consent for publication.

### PEER REVIEW

The peer review history for this article is available at https://publons.com/publon/10.1002/jnr.25098.

## Supporting information

Transparent Science Questionnaire for Authors

## Data Availability

The authors confirm that the data supporting the findings of this study are available within the article.
